# The Impact of Age and Body Composition on Bone Density among Office Worker Women in Hungary

**DOI:** 10.3390/ijerph20115976

**Published:** 2023-05-28

**Authors:** Beáta Vári, Ferenc Győri, Zoltán Katona, Tamás Berki

**Affiliations:** 1 Institute of Physical Education and Sports Science, Faculty of Education, University of Szeged, 6720 Szeged, Hungary; vari.beata@szte.hu (B.V.);; 2 Doctoral School of Health Science, Faculty of Health Science, University of Pécs, 7621 Pécs, Hungary; 3 Institute of Physiotherapy and Sports Science, Faculty of Health Science, University of Pécs, 7621 Pécs, Hungary; 4 Sports Science Research Group, Research Institute, Gál Ferenc University, 6720 Szeged, Hungary

**Keywords:** bone density, body composition, osteopenia, age, Calcaneal Quantitative Ultrasound

## Abstract

The study’s aim was to investigate bone condition and see its associations with body composition and age among office worker women in Hungary. In total, 316 individuals participated in this study from Csongrad-Csanad county in 2019. Participants’ ages ranged from 18 to 62, with a mean of 41 years. A questionnaire was used to gather sociodemographic information, whereas body composition was measured using the Inbody 230, and bone density and bone quality were measured with the SONOST 3000 ultrasound device. Results were analyzed using descriptive statistics, ANOVA with Tukey’s post hoc test, correlation analysis, and an independent sample *t*-test. The results show that Body Fat Mass, Body Mass Index, Obesity Degree, and Percent Body Fat increase significantly as age increases, and Bone Quality Index and *t*-score decrease substantially. Furthermore, Bone Density and Bone Quality Index were positively influenced by most components of body composition. The differences between normal and osteopenia bone quality showed that Basal Metabolic Rate, Bone Mineral Content, Fat-Free Mass, Mineral Mass, Skeletal Lean Mass, and Skeletal Muscle Mass were lower in participants with osteopenia. Our results provide more evidence of the effects of body composition and age on bone density and quality. It was the first study in Hungary investigating this phenomenon, which could be useful for professionals and researchers who intend to understand the associations of bone density.

## 1. Introduction

The battle against chronic diseases is a key topic of recent studies in many fields. The past few decades showed the acceleration of such diseases due to the growing rate of unhealthy lifestyles [1,2,3,4]. Osteoporosis is one of the main concerns that has a growing incidence, and it is associated with low bone density [5]. It is a major problem since it can negatively affect individuals’ quality of life [6]. Bone density decreases with age [5,7,8,9], but an unhealthy lifestyle enhances the risk of the low bone density [10,11,12,13]. Previous studies highlighted gender differences as well. Women are at higher risk for osteoporosis than men since hormonal changes after menopause directly affect bone density [14,15].

The most affected areas in osteoporosis are North America and Europe, but there is a growing trend in developing countries as well [16,17]. Most osteoporotic fractures in disability-adjusted life years (DALY) occurred in Europe (34%), showing a higher prevalence than the most common cancers [18]. In Hungary, the proportion of patients with bone density and bone structure abnormalities increased by 140% between 2011 and 2019, affecting 7–10% of the population [19]. The prevalence of osteoporosis among women over 50 years are four times more common than among men in the European Union. [20].

Unhealthy lifestyle is a major problem in Hungary. Poor diet, sedentary lifestyle, smoking, and alcohol consumption are the main issues, which can be associated with low bone density [21,22,23]. Furthermore, two thirds of the Hungarian population are overweight or obese and generally have higher percentages of body fat and lower percentages of muscle mass compared to other European populations [22]. Due to these problems, the prevalence of osteoporosis in Hungarian women over 50 years of age was 21.6% [24].

Body composition was found as an important predictor for low bone density, but these links have not been investigated well in the Hungarian population. However, other international studies revealed the extensive role of body composition in bone density. For example, a study by Correa-Rodríguez and colleagues [25] found associations between height, weight, Body Mass Index, Lean mass, Body Fat Mass, and Bone Quality Index among young Spanish adults. In another study, Body Fat Percentage and Visceral Fat Percentage were negative predictors of Bone Quality Index [26]. Other studies also indicated that high level of Body Fat decreases bone quality among sedentary worker women [12,27,28]. Interestingly, in a study of more than 6000 Italian women aged 30–80 years, obesity was found to be a protective factor against osteoporosis [29]. Consistent with these findings, high Bone Mineral Density was found to be associated with increased Body Mass Index and waist circumference [30,31,32]. Brunner and colleagues [7] studied the elderly German population and found that Fat-Free Mass had a positive influence on bone quality in the heel. Gjesdal [33] came to similar conclusions when examining the relationship between Fat-Free Mass and Bone Mineral Density at the femoral neck. Muscle mass has been identified as a protective factor for bone health, as a positive association between muscle mass and Bone Mineral Density has been found [14,34]. According to Jiang and colleagues [34], lower limb muscle mass is better at preventing osteoporosis than upper limb skeletal muscle.

Overall, several body composition factors have a role in bone density, but the studies show some inconsistencies. Therefore, investigating this issue from another perspective would be beneficial. Furthermore, recent Hungarian studies on this topic mainly investigated the relationship between unhealthy lifestyle and bone density, and there is a lack of information on the body composition of Hungarian women [35,36]. Therefore, the goal of this study is to analyze bone conditions and see their associations with body compositions and ages among office worker women. We analyzed this population since they have an increased risk of undesirable body compositions and lower bone densities [12,37]. We hypothesize that the values of body composition components (e.g., BMI, Fat Mass, Bone Mineral Content, etc.) are related to bone density and bone quality. In addition, we assume that bone density, bone quality, and body composition will deteriorate with age.

## 2. Materials and Methods

### 2.1. Participants and Procedure

Participants in this study were recruited among office worker women in the Fall of 2019. They were employees for the local government office in Csongrad-Csanad county, Hungary. All subjects agreed to participate in this study. They were assured no personal data (e.g., names) would be collected, and all data would be used for statistical purposes. They had to take an online-based questionnaire before they came to the face-to-face physical examinations. A total of 316 participants aged between 18–62 was willing to participate in this study (Age_Mean_ = 41; Age_SD_ = 9). For the study purposes and to make visible changes for the observed variables, the sample was divided into four age groups following the previous literature [1,38,39]: young adults—aged 18–29 (*n* = 50); adults—aged 30–39 (*n* = 88); middle-aged adults—aged 40–49 (*n* = 104); older adults—above 50 (*n* = 74).

After, the participants filled out the short questionnaire about their socio-demographics. Bone density was measured using Quantitative ultrasound (QUS) with SONOST 3000, and body composition was measured using Inbody 230. The whole process was supervised by the same trained medical team. The investigation lasted three weeks and was conducted in the central building of the Szeged government office. They came to the designated room at a scheduled time every day. As 88 questionnaires had already been completed, only the physical examinations were performed. First, body height, body composition, and, finally, bone density were measured. In each case, the measurement parameters of the right calcaneus (heel bone) were recorded. The bone examination and body analysis took approximately 15 min for each subject. The study was approved by the institutional ethical board (ethical code: 2/2019SZTE).

### 2.2. Measures

#### 2.2.1. Sociodemographic Questions

Participants were asked about their sociodemographic data to see the sample’s characteristics. The questionnaire contained close-ended questions on their age, education, and family background (e.g., “What is your highest education?”).

#### 2.2.2. Body Composition

Body composition was measured with Inbody 230 (Biospace Co. Ltd., Seoul, Republic of Korea). The participants were asked to stand on the device barefoot and hold the thumb electrode for 30 s. The following parameters were calculated by this device: Abdominal Obesity Degree, Basal Metabolic Rate, Body Fat Mass Bone Mineral Content, Fat Free Mass, Mineral Mass, Obesity Degree. Percent Body Fat, Skeletal Lean Mass, Skeletal Muscle Mass, Visceral Fat Area, and Body Mass Index. Table 1 represents all the definitions and units of measures [40,41,42,43].

#### 2.2.3. Bone Density and Bone Quality

Bone Density and Bone Quality Indexes were measured with a SONOST 3000 (Osteosys, Seoul, Republic of Korea) ultrasound bone densitometer. SONOST 3000 is a mobile Bone Mineral Density meter that is used to measure broadband ultrasound attenuation (BUA) and the speed of sound (SOS), which are both related to temperature. It is a non-invasive and portable device commonly used in clinical settings for bone density screening and assessment [44,45]. The SONOST 3000 was calibrated daily using according to the manufacturer’s instructions to ensure the accuracy of the measurements [46]. The device is primarily used to assess bone quality by measuring the *t*-score and Bone Quality Index at the calcaneus of the right or the left heel. Quantitative Ultrasound were found to be an effective method to predict fracture and Bone Mineral Density, and it could reduce the number of dual-energy X-ray absorptiometry [5,20,47].

The *t*-score of the right heel was determined using the operations manual of the equipment [46]. The *t*-score is output directly by the ultrasound device, and it is calculated by comparing a person’s lower bone density scores, indicating a greater risk of fracture and a lower level of bone density. The classification provided by the WHO [48] was used to determine normal, osteopenia, and osteoporosis levels of bone density. Hence, a T-score value above –1 was classified as normal. *t*-scores of −1 and −2.5 were classified as moderate or osteopenia bone densities, and *t*-scores below −2.5 were classified as low or osteoporosis bone densities [20].

Bone Quality Index was calculated as well. Bone Quality Index represented the bone strength of the individuals. The accuracy error of Bone Quality Index was less than 1.5%. There was no exact reference range of Bone Quality Index since the process depends on the specific ultrasound device. SONOST 3000 calculates Bone Quality Index as follows: BQI = αSOS + βBUA (αβ: temperature correction) [46]. The higher Bone Quality Index score indicates better bone quality and lower fracture risk. Bone Quality Index values ranged between 11.3 and 128 in this study.

### 2.3. Statistical Methods

Descriptive statistics were used to determine the characteristics of the sample. Kolmogorov–Smirnov test was used to see the normality of our data. The test showed that our variables did not deviate significantly from a normal distribution. Hence, One-way analysis of Variance (ANOVA) with Tukey’s post hoc test was used to understand the differences between the body composition, *t*-score, and Bone Quality Index in different age groups. The homogeneity of variance was examined with Leven’s test. The homogeneity assumption of the variance was met since all variables showed non-significant results. For the comparison of age, *t*-score, Bone Quality Index, and body composition, correlation analysis was utilized. Age was examined using two different methods for better understanding. Finally, Independent Sample *t*-test was used to determine the significant differences between normal bone quality and osteopenia bone quality. Eta square (η²) and Cohen’s d were used as effect sizes for ANOVA and *t*-test. Eta-squared values ranged from 0 to 1, with larger values indicating a stronger relationship between the variables. Cohen’s d value of 0.2 was considered a small effect size, 0.5 was a moderate effect size, and 0.8 or higher was a large effect size. SPSS for Windows 15.0 was used for all statistical analysis. The significance level was set at 95% in all cases.

## 3. Results

Table 2 shows the components of the Body Composition, T-score, and Bone Quality Index between age groups. Abdominal Obesity Degree (F = 5.96; *p* < 0.001), Body Fat Mass (F = 7.13; *p* < 0.001), Body Mass Index (F = 8.44; *p* < 0.001), Obesity Degree (F = (8.43; *p* < 0.001), Percent Body Fat (F = 9.84; *p* < 0.001), Visceral Fat Area (F = 11.86; *p* < 0.001), Bone Quality Index (F = 4.86; *p* < 0.05), and *t*-score (F = 148.71; *p* < 0.001) were found to be significant. The Tukey post hoc test revealed that the age groups between 18–29 were significantly (*p* < 0.05) lower than the age groups of 30–49 and 50+ in the variable of Abdominal Obesity Degree, Body Mass Index, Obesity Degree, Percent Body Fat, and Visceral Fat Area. Body Fat Mass, Bone Quality Index, and *t*-score were only significantly differentiated between age groups of 18–29 and 50+. Even though the post hoc test showed the main significant differences between the age groups, the trend showed a deterioration with the incasement of age in all the variables.

Correlation analysis showed that age had significant and positive associations between Abdominal Obesity Degree (r = 0.22), Body Fat Mass (r = 0.28), Body Mass Index (r = 0.31), Obesity Degree (r = 0.32), Percent Body Fat (r = 0.33), and Visceral Fat Area (r = 0.22). Bone Quality Index was positively associated with Basal Metabolic Rate (r = 0.18), Body Fat Mass (r = 0.14), Body Mass Index (r = 0.15), Bone Mineral Content (r = 0.17), Fat Free Mass (r = 0.18), Mineral Mass (r = 0.17), Obesity Degree (r = 0.15), Skeletal Lean Mass (r = 0.18), and Skeletal Muscle Mass (r = 0.18). The associations of *t*-score were the same as Bone Quality Index. Thus, Basal Metabolic Rate (r = 0.20), Body Fat Mass (r = 0.14), Body Mass Index (r = 0.16), Bone Mineral Content (r = 0.19), Fat Free Mass (r = 0.20), Mineral Mass (r = 0.19), Obesity Degree (r = 0.16), Skeletal Lean Mass (r = 0.20), and Skeletal Muscle Mass (r = 0.20) were positive predictors of the components of body composition (Table 3).

Finally, we categorized the participants using T-score into normal and osteopenia (lower bone density) bone quality, then we analyzed the body composition differences between the two groups. It should be noted again that there was no individual with osteoporosis bone quality. Table 4 shows that Basal Metabolic Rate (*t* = 1.98; *p* < 0.01) were significantly higher for normal bone quality. Fat Free Mass (*t* = 1.98; *p* < 0.05), Mineral Mass (*t* = 1.99; *p* < 0.05), Skeletal Lean Mass (*t* = 1.97; *p* < 0.05), and Skeletal Muscle Mass (*t* = 1.30; *p* < 0.05) were found to be higher for normal bone quality.

## 4. Discussion

The aim of the study was to understand the relationship between age, body composition, bone density, and bone quality among office worker women in Hungary. To the best of our knowledge, this was the first study investigating this population. We also intended to increase the number of publications about the relationship between bone density and body composition in Hungary.

Two methods were used to examine the relationship between age, body composition, Bone Density, and Bone Quality Index to understand the complex associations between these variables. Even though we hypothesized that body composition worsened for the older age groups, our results only suggested that the components of body composition that increase with age were related to factors associated with obesity [49]. These associations were also consistent with previous studies [29,30,31,32], and there was a clear trend that showed increased Body Mass Indexes in the older women population in Hungary [22]. Other components that described muscle structure, metabolism, and mineral mass did not show significant differences with age in this study. Our results seemed to support the findings that atrophy and mineral loss primarily affect people older than 70 years [50,51]. Basal Metabolic Rate did not differ with age as well, but it is important to note that Free Fat Mass, which is linked to Basal Metabolic Rate, did not differentiate either between the age groups [52].

Bone density and the Bone Quality Index were found to be lower in older age groups than in younger age groups. The association is well-known among researchers and corresponded to our hypotheses [5,7,9,14]. According to the literature, people over the age of 50 are more likely to suffer from bone loss [53], which was reflected in our findings. Women are at a higher risk than men due to hormonal changes. Thus, physical activity and a healthy diet are more important for this population [10,11,15].

There is a lack of studies investigating the relationship between body composition, Bone Density, and Bone Quality Index in the Hungarian population. It turned out in this study that the components related to muscle mass (e.g., Skeletal Muscle Mass, Skeletal Lean Mass) were found to increase bone density and bone quality while also protecting against osteopenia. Although there appears to be a clear relationship between these variables, studies on the role of muscle mass on bone condition are inconsistent. For example, Jiang and colleagues [34] identified as a protective factor on bony condition, whereas Brunner [7] discovered an inverse relationship. Components related to obesity also showed a positive role on Bone Density and Bone Quality Index. Several previous studies found similar relationships [32,54,55]. The reason for this phenomenon is that increased body mass indicates increased mechanical load, which may improve bone structure [56].

Our findings also indicated that bone mineral content and mineral mass were beneficial to bone density and bone quality, as higher mineral content in the bones indicated greater bone strength [57,58]. It was also higher in people with normal bone health. As a result, promoting a healthy diet is critical because it can increase bone minerals [11,17,25]. The Basal Metabolic Rate was also positively correlated to bone quality and density, and it was significantly higher for individuals with normal bone conditions. Choi and Pai [59] discovered a similar result. According to their findings, bone density was highly correlated with BMR in postmenopausal women. However, they also found Basal Metabolic Rate values higher for women with osteoporosis. We believe these associations may have been more complex; therefore, further research into this phenomenon is required, and the key might be the diet of the Hungarian population.

The study had some limitations that should be addressed. At first, we should underline that this study only included women and did not investigate the effects of menopause on bone density; we did not have the opportunity to see hormonal and other metabolism changes during this study. Furthermore, we should emphasize that the subgroup’s sample was not equal, and the data were collected in only one county in Hungary. Thus, our findings could not be generalized to the entire Hungarian population. Another issue was that we did not include lifestyle factors (e.g., physical activity, diet) that could affect bone density. In the future, we hope to continue collecting data and expand the study to a national scale even by using an osteoporosis-specific questionnaire [60] as a supplement to the instrumental measurements. Our future goal is to add other factors (e.g., working conditions) and understand the risk factors (e.g., physical inactivity) of low bone conditions among Hungarian women. This study has shown great interest from our participants since the feedback they receive helps them raise their awareness of a healthier lifestyle that could prevent osteopenia or osteoporosis.

## 5. Conclusions

In conclusion, our study confirms the hypothesized relationship between body composition, age, bone quality, and bone density. Our findings lead us to the following conclusions: (1) Age has a negative impact on the components of body composition that are linked to obesity; (2) Bone Density and Bone Quality Index are decreasing with age; (3) Increasing muscle and mineral mass help to prevent osteopenia; (4) Obesity-related factors seems to have a protective effect on bone density and bone quality, but our results are inconsistent. Thus, physical activity should be promoted even with overweight individuals since it could prevent bone loss in older ages. We hope that we can have a more detailed picture of Hungarian women’s bone conditions and body compositions that could help to suggest new programs for maintaining the condition of this population with our study.

## Figures and Tables

**Table 1:** Description of measured variables of body composition.

Variable	Definition
Abdominal Obesity Degree (W/H)	The abdominal Obesity Degree is the ratio of waist and hip circumference. Abdominal obesity is diagnosed in cases of over 0.90 for males and 0.85 for females.
Basal Metabolic Rate (Kcal)	The Basal Metabolic Rate is the minimum energy requirement that the body needed to sustain vital functions while at rest.
Body Fat Mass (Kg)	Body fat mass refers to the amount of fat in the body. Body Fat Mass is the sum of subcutaneous fat, visceral fat, and fat surrounding muscles.
Body Mass Index (Kg/m^2^)	Body mass index is a measure of body fat based on an individual’s weight and height. It is calculated by dividing a person’s weight in kilograms (kg) by their height in meters squared (m²).
Bone Mineral Content (Kg)	Bone Mineral Content is the weight of minerals in bone.
Fat Free Mass (Kg-BFM)	Fat Free Mass is the weight of everything except body fat. This includes muscle, water, bones, organs—everything that is not body fat.
Mineral Mass (Kg)	Minerals refer to the total amount of inorganic minerals that are dissolved in bone and body fluids that represent osseous and non-osseous minerals, respectively.
Obesity Degree (%)	Obesity Degree is the ratio of current weight to ideal weight. Obesity Degree = (current weight/standard weight by height) × 100.
Percent Body Fat (%)	Percent body fat is a measure of the amount of fat in the body as a percentage of total body weight. It is calculated by dividing the weight of body fat by the total body weight and multiplying it by 100.
Skeletal Lean Mass (Kg)	Skeletal lean mass refers to the lean muscle and bone tissue found in the skeleton. It is the total amount of muscle, bone, and connective tissue in the body that is not composed of fat.
Skeletal Muscle Mass (Kg)	Skeletal muscle mass, which generally indicates the lean body mass of each arm and leg.
Visceral Fat Area (cm²)	Visceral Fat Area is the estimated area of fat surrounding internal organs in the abdomen. A Visceral Fat Area under 100cm² is the healthy range.

**Table 2:** Age related differences on Body Composition and Bone Density.

	18–29 (M, SD) *n* = 50	30–39 (M, SD) *n* = 88	40–49 (M, SD) *n* = 104	50+ (M, SD) *n* = 74	F	η^2^	*p* Value
Abdominal Obesity Degree (W/H)	0.88 (0.06)	0.90 (0.07)	0.92 (0.06)	0.93 (0.06)	5.96	0.05	0.001
Basal Metabolic Rate (Kcal)	1342.56 (151.75)	1359.86 (126.12)	1375.85 (137.63)	1363.41 (138.18)	0.62	0.01	0.634
Body Fat Mass (Kg)	20.93 (9.26)	23.46 (10.88)	25.72 (10.82)	29.42 (12.13)	7.13	0.07	0.001
Body Mass Index (Kg/m^2^)	23.82 (4.69)	25.05 (5.23)	26.70 (5.52)	28.31 (6.21)	8.44	0.08	0.001
Bone Mineral Content (Kg)	2.69 (0.43)	2.70 (0.34)	2.72 (0.37)	2.66 (0.35)	0.46	0.00	0.725
Bone Quality Index	87.76 (17.48)	83.56 (15.08)	81.04 (15.83)	76.95 (15.25)	4.86	0.04	0.016
Fat Free Mass (Kg-BFM)	45.02 (7.03)	45.83 (5.83)	46.57 (6.37)	45.99 (6.41)	0.62	0.01	0.634
Mineral Mass (Kg)	3.23 (0.52)	3.25 (0.41)	3.28 (0.45)	3.21 (0.42)	0.41	0.00	0.771
Obesity Degree (%)	110.78 (21.82)	116.54 (24.32)	124.14 (25.68)	131.72 (28.89)	8.43	0.08	0.001
Percent Body Fat (%)	30.64 (7.26))	32.42 (8.29)	34.40 (7.67)	37.65 (7.96)	9.84	0.09	0.001
Skeletal Lean Mass (Kg)	42.33 (6.61)	43.12 (5.50)	43.85 (6.02)	43.33 (6.09)	0.67	0.01	0.597
Skeletal Muscle Mass (Kg)	24.63 (4.21)	25.11 (3.50)	25.56 (3.82)	25.16 (3.82)	0.63	0.01	0.633
*t*-score	−0.93 (0.94)	−1.15 (0.81)	−1.24 (0.79)	−1.51 (0.82)	148.71	0.04	0.019
Visceral Fat Area (cm²)	87.46 (35.80)	97.97 (41.83)	110.12 (39.04)	126.32 (41.11)	11.86	0.10	0.001

**Table 3:** Correlation analysis of Body Composition, Age, and Bone Quality Index.

	Age	Bone Quality Index	*t*-Score
Abdominal Obesity Degree (W/H)	0.22 (*p* = 0.001)	0.03 (*p* = 0.506)	0.04 (*p* = 0.452)
Basal Metabolic Rate (Kcal)	0.06 (*p* = 0.259)	0.18 (*p* = 0.002)	0.20 (*p* = 0.001)
Body Fat Mass (Kg)	0.30 (*p* = 0.001)	0.14 (*p* = 0.016)	0.14 (*p* = 0.014)
Body Mass Index (Kg/m^2^)	0.31 (*p* = 0.001)	0.15 (*p* = 0.007)	0.16 (*p* = 0.004)
Bone Mineral Content (Kg)	−0.02 (*p* = 0.725)	0.17 (*p* = 0.004)	0.19 (*p* = 0.001)
Fat Free Mass (Kg-BFM)	0.06 (*p* = 0.260)	0.18 (*p* = 0.002)	0.20 (*p* = 0.001)
Mineral Mass (Kg)	0.01 (*p* = 0.918)	0.17 (*p* = 0.03)	0.19 (*p* = 0.001)
Obesity Degree (%)	0.32 (*p* = 0.001)	0.15 (*p* = 0.007)	0.16 (*p* = 0.004)
Percent Body Fat (%)	0.33 (*p* = 0.001)	0.08 (*p* = 0.166)	0.08 (*p* = 0.172)
Skeletal Lean Mass (Kg)	0.07 (*p* = 0.223)	0.18 (*p* = 0.002)	0.20 (*p* = 0.001)
Skeletal Muscle Mass (Kg)	0.06 (*p* = 0.294)	0.18 (*p* = 0.001)	0.20 (*p* = 0.001)
Visceral Fat Area (cm²)	0.22 (*p* = 0.001)	0.09 (*p* = 0.098)	0.10 (*p* = 0.087)

**Table 4:** Body Composition differences between normal and osteopenia bone quality.

	Normal (M, SD) n = 190	Osteopenia (M, SD) n = 126	*t*-Value	Cohen’s d	*p* Value
Abdominal Obesity Degree (W/H)	0.92 (0.07)	0.91 (0.07)	1.14	0.12	0.315
Basal Metabolic Rate (Kcal)	1378.34 (148.19)	1344.60 (115.60)	2.13	0.25	0.034
Body Fat Mass (Kg)	25.96 (11.71)	24.05 (10.45)	1.48	0.18	0.131
Body Mass Index (Kg/m^2^)	26.58 (5.86)	25.53 (5.36)	1.62	0.20	0.089
Bone Mineral Content (Kg)	2.73 (0.40)	2.65 (0.32)	2.09	0.24	0.038
Fat Free Mass (Kg-BFM)	46.55 (6.86)	45.12 (5.36)	2.13	0.25	0.034
Mineral Mass (Kg)	3.29 (0.47)	3.19 (0.38)	2.12	0.25	0.035
Obesity Degree (%)	123.63 (27.26)	118.75 (24.93)	1.61	0.20	0.091
Percent Body Fat (%)	34.39 (8.15)	33.44 (8.21)	1.01	0.11	0.339
Skeletal Lean Mass (Kg)	43.82 (6.49)	42.47 (5.06)	2.13	0.25	0.034
Skeletal Muscle Mass (Kg)	25.54 (4.12)	24.64 (3.19)	2.18	0.25	0.030
Visceral Fat Area (cm²)	109.19 (42.09)	103.19 (41.23)	1.96	0.15	0.210

## Data Availability

The data presented in this study are available on request from the corresponding author.
